# Associations between childhood maltreatment and adult depression: a mediation analysis

**DOI:** 10.1186/s12888-019-2016-8

**Published:** 2019-01-22

**Authors:** Anne Klumparendt, Janna Nelson, Jens Barenbrügge, Thomas Ehring

**Affiliations:** 1 Department of Psychology, University of Münster, Fliednerstr. 21, 48149 Muenster, Germany; 2 Christoph-Dornier-Foundation for Clinical Psychology, Münster, Germany; 3 Department of Psychology, LMU Munich, Munich, Germany

**Keywords:** Childhood Maltreatment, Risk Factor, Depression, Psychological Mediators

## Abstract

**Background:**

There is ample evidence showing that childhood maltreatment (CM) is a risk factor for the development of depression in adulthood. However, little is known about the psychological processes mediating this relationship. This study used a large community sample to investigate the mediating role of emotional, cognitive and/or interpersonal dysfunctions on the one hand and posttraumatic stress disorder symptoms on the other hand.

**Methods:**

One thousand twenty seven participants of a community sample filled out an online survey. Mediation analyses were computed via linear structural equation modelling.

**Results:**

Results showed a significant mediation of the association between CM and adult depression via emotional impairments, depressogenic attribution style and symptoms of posttraumatic stress disorder. Our study design was cross-sectional and therefore did not allow testing temporal precedence of mediators and causality. Data was collected retrospectively, a confounding effect of current depressive symptoms on retrospective recall of CM therefore cannot be ruled out.

**Conclusions:**

The a priori mediation model showed a good fit with the data. The model suggests promising objectives for further research on CM-related depression and potential treatment targets in the future.

## Background

Childhood maltreatment (CM), defined as sexual, physical and/or emotional abuse and/or physical or emotional neglect [1, 2], has been shown to be an important risk factor for major depressive disorder (MD) in adulthood [3]. Importantly, CM is not only related to an increased risk for MD, but also its course [4, 5]. More precisely, the experience of any form of CM more than doubles the risk of developing a MD, and is associated with an earlier onset, higher symptom severity and more episodes; moreover, CM survivors will more likely be non-responders to common treatments (psychological treatment, pharmacotherapy or their combination) [4, 5]. Improving treatment for CM survivors suffering from depression is therefore an important clinical challenge. To this aim, it appears necessary to gain a better understanding of the processes mediating CM and MD, which could be the groundwork for developing psychological interventions directly targeting the mechanisms that underlie MD in CM survivors.

### Mediators

Earlier research has identified candidates for biological processes mediating CM and MD, such as neuroendocrine changes that reflect sensitization of central stress response systems [6, 7], neuroanatomical and neurofunctional changes leading to emotional and/or cognitive dysfunction [8], and alterations in the inflammatory system [9]. However, much less is known about *psychological* mediators, despite the fact that these may be promising targets for psychological treatment. The current study focused on four groups of potential psychological mediators between CM and MD, namely emotional regulation difficulties, attachment, attributional style, and symptoms of posttraumatic stress disorder.

#### Emotion regulation difficulties

Emotion regulation (ER), defined as “processes through which individuals modulate their emotions consciously or unconsciously to appropriately respond to environmental demands” ([10], p. 218), play an important role for a person’s mental health [11, 12]. Difficulties in ER are conceptualized both as an impairment of general regulation processes (e.g. acceptance and capacity to engage in goal-directed behavior when distressed; [13]) and as the use of dysfunctional ER strategies [10]. ER is usually developed during childhood in interaction with important caregivers [12]. This development can be impaired in an environment of abuse and neglect, especially when the primary caregiver is the abuser [14]. Evidence comes from studies showing that CM leads to difficulties in recognizing, understanding and regulating emotions [15–17]. Difficulties in ER are in turn associated with different types of psychopathology, including MD [10, 12]. Excessive rumination is one particularly dysfunctional ER strategy that has been shown to be associated with the development and maintenance of MD [18, 19]. Importantly, some recent studies have provided preliminary evidence showing that ER difficulties mediate the association between CM and MD [20–22]. While Hopfinger et al. [20] and Huh et al. [21] computed mediation analyses with ER as a single mediator, Schierholz et al. [22] tested a multiple mediation model showing that ER still mediated between CM and MD when other variables such as depressogenic attributional style or attachment-related avoidance were controlled.

#### Attachment

On the basis of Brennan et al.’s [23] seminal approach, adult attachment is often described on the two dimensions of attachment-related anxiety and avoidance [24]. People who score low on these dimensions show a secure attachment orientation, while instead high scores on one or both dimensions reflect an insecure attachment style [23]. There is strong evidence showing that CM impacts on early attachment relationships and is related to insecure (anxious and avoidant) attachment patterns later in life [25–27]. In addition, insecure attachment is associated with risk for depression [28, 29]. In their review of the literature, Riso et al. [30] suggest that adult interpersonal problems as a result of disrupted attachment representations may mediate the association between negative experiences during childhood and chronic depression. To our knowledge, only one study to date has directly tested whether attachment statistically mediates between CM and MD [22]. Results showed a significant indirect effect of avoidance in close relationship on the association of CM and the number of depressive episodes [22].

#### Attributional Style

Early interpersonal trauma can be seen as an extreme form of negative and uncontrollable life events [31] and therefore prototypical to lead to dysfunctional cognitive schemes like helplessness and worthlessness [32]. In particular, being exposed to repeated or chronic CM may lead to a tendency to attribute negative events to internal, stable and global causes [33]. This so-called depressogenic attributional style forms the core of the hopelessness theory of depression postulated by Abramson et al. [34], which describes a higher risk of MD for individuals with this attributional style [33]. Wiersma et al. [7] could show that a more chronic course of depression is associated with a higher level of hopelessness and an external locus of control [7]. First evidence for a mediational effect of depressogenic attributional style on the relationship between CM and MD was found by Schierholz et al. [22], who showed significant indirect effects of CM via depressogenic attributions on both the number of depressive episodes and depressive symptom severity.

#### Posttraumatic Stress Disorder (PTSD)

Psychological disorders other than MD are highly prevalent in CM survivors, especially PTSD [35]. PTSD has been shown to have a strong negative impact on functioning [36], and often leads to secondary symptoms of MD [37]. It is therefore conceivable that PTSD symptoms may be an additional mediator between CM and MD. In an earlier study on 340 depressed individuals, Schierholz et al. [22] found preliminary evidence supporting this view. In a first multiple mediation model, they found that the three variables described above, emotion regulation difficulties, a depressogenic attributional style, and avoidance in close relationships as a facet of insecure attachment, conjointly mediated the relationship between CM and depression. However, a significant direct path between CM and MD symptoms remained in this model. In a subsequent multiple mediation model, PTSD symptom severity was entered as an additional mediator, leading to full mediation between CM and MD.

However, as PTSD symptom scores were not available for all participants, the second mediation model could only be run in a subsample, which renders these findings preliminary. In addition, although Schierholz et al. [22] modeled PTSD symptom severity as an independent mediator between CM and depression, emotion regulation difficulties, negative attributional style, and insecure attachment have all been shown in earlier research to be related to PTSD (e.g. [16, 38, 39]). Therefore, in addition to direct paths from these variables to depression it can be hypothesized that these variables additionally impact on depression through an indirect path via PTSD symptom severity.

### The current study

**Fig. 1:**
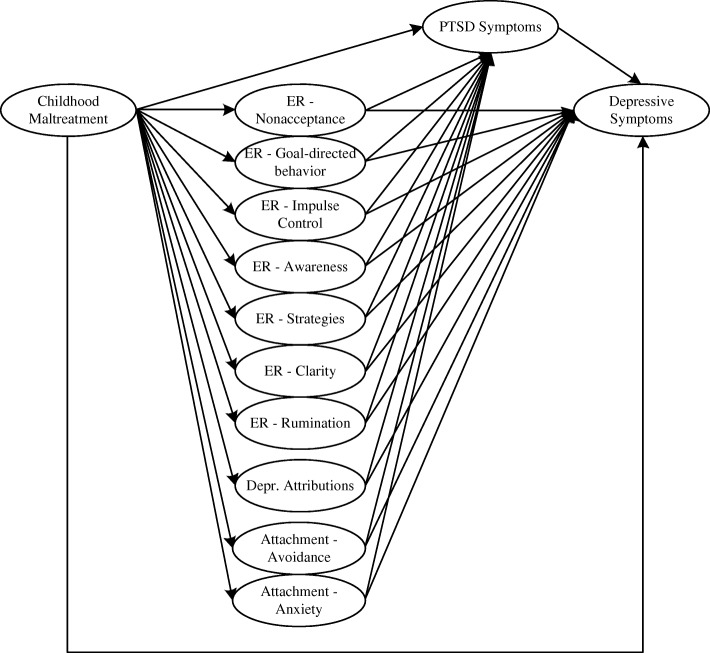
Postulated Mediational Path Model. For reasons of clarity and comprehensibility, this is only a simplified version of the calculated model. Measurement models of the latent constructs and also residual correlations are not displayed. ER = emotion regulation

The current study aimed to test a sequential mediator model to account for the association between CM and MD symptoms. Emotion regulation difficulties, insecure attachment, depressogenic attributional style, and PTSD symptom severity were included as four different groups of mediators, whereby the former three were modeled to not only directly impact on MD symptoms but also show an additional indirect path via PTSD symptom severity (see Fig. 1 for a simplified illustration of the mediation model).

The study is based on earlier research investigating these mediators. However, we aimed to extend these findings in important ways. First, whereas earlier studies have tended to examine these mediators in isolation, this study tests a comprehensive model including several postulated mediators simultaneously. Second, our model includes PTSD symptom severity as a mediator, which has been neglected in most earlier studies. Third, latent modelling of all variables of interest was used to provide more accurate estimations. Finally, whereas most earlier studies tested clinical samples with somewhat reduced variance on the variables of interest, the current study focused on a population sample with a large range of symptom severities ranging from no symptoms at all to severe levels of depression.

Our main hypothesis was that the sequential mediation model would show a good model fit (Hypothesis 1). In addition, we expected a significant total indirect effect and a non-significant direct effect in our model (Hypothesis 2). Finally, we expected specific indirect effects to include PTSD symptom severity (Hypothesis 3a), and variables indicative of impaired emotional, cognitive, and interpersonal development with a direct path to MD symptoms (Hypothesis 3b) as well as an indirect path from these variables to MD symptoms via PTSD symptom severity (Hypothesis 3c).

## Methods

### Participants

Participants were recruited via PsyWeb (https://psyweb.uni-muenster.de/), a non-commercial online panel for individuals from the general population who are interested in supporting psychological research. E-mails were sent to every registered user of PsyWeb (approximately 12,000 individuals). The e-mails included general information about the study and a link that led to the online questionnaire. 1,966 persons initially retrieved the first page. Inclusion criteria were: (1) age between 18 and 65 years, (2) fluent in German, (3) no bipolar and/or psychotic symptoms, and (4) no acute suicidality. 271 participants were excluded as they did not meet inclusion criteria, 635 discontinued the survey before completion, 12 did not provide informed consent^1^, and 21 were excluded because of too much missing data (>50%) in the CTQ^2^. This resulted in a final sample of 1,027 participants to be analyzed (age: *M* = 45.1, *SD* = 11.89, age range: 18-65; 68.6% female).

### Design and Procedures

This cross-sectional study was conducted as an online survey offered via Unipark (http://www.unipark.com/). Independent and dependent variables as well as proposed mediators were assessed using self-report questionnaires. The study was approved by the local research ethics committee.

### Instruments

#### Childhood Maltreatment

Experience of maltreatment during childhood (CM) was assessed using the German version of the Childhood Trauma Questionnaire (CTQ; [40]; German version: [41]), which is a reliable and valid instrument for the retrospective assessment of childhood maltreatment [42]. The CTQ comprises 28 items, forming five subscales of five items each (*physical, sexual, emotional abuse and physical, emotional neglect*; e.g. “Someone tried to touch me in a sexual way or tried to make me touch them.”). Three additional validity items measure the tendency to trivialize one’s experiences (e.g. “I had the perfect childhood.”) and are not used in analyses. Every item is rated on a 5-point Likert-scale (1 = *never true* to 5 = *very often true*). To aggregate the data for our calculations, we modeled a general factor (CM) defined by five first-order latent factors. The five first-order factors matched the postulated factor structure of Bernstein et al. [40] except from one modification: the item “When I was growing up, I knew there was someone there to take care of me and protect me” was set as indicator of the factor *emotional neglect* instead of *physical neglect*. Fit indices indicated an excellent model fit for the general factor model, χ^2^(270) = 1061.13, *p* < .001, RMSEA = .05, CFI/TLI = .99/.98.

#### Depression

The severity of current depressive symptoms was assessed with the depression module of the Patient-Health Questionnaire (PHQ-9; [43]; German version: [44]). It consists of 9 items (e.g. “Little interest or pleasure in doing things.”) that are rated on a scale ranging from 0 (*not at all*) to 3 (*nearly every day*). Gräfe et al. [45] found high criterion-related validity of the German version with a specificity of 86% and a sensitivity of 95% when SCID diagnoses were used as the gold standard criterion. In addition to screening for the presence of MD, the PHQ-9 sum score can also be used dimensional to index MD symptom severity. A sum score of 5 or above is indicative of at least mild symptoms of depression [43, 45]. In our analyses, we modeled one single latent factor of MD defined by all PHQ-9 items.

#### Emotion Regulation Difficulties

ER is a heterogeneous concept, including both impaired general processes and dysfunctional strategies [10, 13], and there is currently no universally accepted taxonomy. Therefore, we decided to include not only a single factor for emotion regulation in our mediation model, but several latent variables assessed with two different instruments. The Difficulties in Emotion Regulation Scale (DERS; [13]; German version: [46]) comprises 36 items falling onto the six subscales *nonacceptance of emotional responses*, *difficulties engaging in goal-directed behavior*, *impulse control difficulties*, *lack of emotional awareness*, *limited access to emotion regulation strategies* and *lack of emotional clarity*. Items are scored on a 5-point Likert-scale (1 = *almost never* to 5 = *almost always;* e.g. “I am confused about how I feel”). The six-factor structure was replicated for the German version with good psychometric properties for all subscales (internal consistencies ranging between .76 and .87; [46]). Our measurement model corresponds to this six-factor structure.

Depressive rumination as a specific dysfunctional regulation strategy was assessed with the brooding subscale of Response Style Questionnaire (RSQ-10D; [47]; German version: [48]). The subscale consists of five items asking what participants do when they feel down, sad or depressed (e.g. “Think ‘What am I doing to deserve this?’”). Items are scored on a 4-point Likert-scale (1 = *almost never* to 4 = *almost always*). Considering the small number of items, Huffziger and Kühner [48] found acceptable internal consistencies (α = .60 – .70). Negative associations with subjective well-being and positive associations with depression further demonstrate the construct validity [48]. In line with these findings, we operationalized the tendency to ruminate as a single factor with the five items of the rumination subscale as indicators.

#### Attributional Style

The 16-item Depressive Attributions Questionnaire (DAQ; [49]; German version: [22]) was used to measure depressogenic attributional styles, namely the tendency to attribute negative life events to internal, stable and global causes (e.g. “When bad things happen, I think it is my fault.”). Items are rated on a scale between 0 = *not at all* and 4 = *very strongly*. Exploratory factor analyses revealed good fit-indices (RMSEA = .09, CFI/TLI = .91/.92) and internal consistencies (α = .94 - .97) for the one-dimensional structure [49]. Schierholz et al. [22] found excellent internal consistencies (α = .93) for the German version. Our measurement model adopted the originally postulated one-dimensional structure [49].

#### Attachment

In order to assess the quality of adult attachment on the two dimensions *anxiety* and *avoidance*, participants also filled in the German version of the Experiences in Close Relationship Scale (ECR; [23]; German version: [50]). The questionnaire consists of 36 items (response scale ranging from 1 = *strongly disagree* to 7 = *strongly agree*; e.g. “I get uncomfortable when a romantic partner wants to be very close.”), forming two subscales of 18 items each. Neumann et al. [50] confirmed the two-factor-structure and reported good internal consistencies for each subscale (Cronbach’s α ranging between .85 and .91). For our analyses, we adopted this two-factor-structure.

#### Posttraumatic Stress Disorder

Symptoms of Posttraumatic Stress Disorder were assessed using the 20-item PTSD-Checklist for DSM-5 (PCL-5; [51]; German version: [52]). The PCL-5 corresponds to the DSM-5-criteria for PTSD [53] and assesses the four symptom cluster *re-experiencing* (items 1-5), *avoidance* (items 6-7), *negative alterations in cognitions and mood* (items 8-14) and *alterations in arousal and reactivity* (items 15-20). Items are rated on a 5-point Likert-scale ranging from 0 = *not at all* to 4 = *extremely* (e.g. “Repeated, disturbing and unwanted memories of the stressful event.”). Recent results regarding the German version indicate good psychometric properties with high internal consistency for a total symptom score (Cronbach’s α = .95; [52]). In line with these results, we modeled a second-order general-factor model representing PTSD symptoms. To avoid substantial content overlap with the dependent variable MD symptoms and hereby biased mediational effects of PTSD symptoms, we defined this general-factor solely by the three first-order factors, *re-experiencing* (items 1-5), *avoidance* of trauma-associated stimuli (items 6-7) and *hypervigilance* (items 17-18), which represent exclusively PTSD symptoms. As there is no empirical evidence for the goodness of fit of this model yet, a CFA was calculated and revealed good fit indices, χ^2^(24) = 109.47, *p* < .001, RMSEA = .06, CFI = .99, TLI = .99.

### Data Analysis

The analysis of descriptive data was conducted via IBM SPSS Statistics, version 22. Correlation coefficients between all latent constructs of interest were estimated using structural equation modelling (SEM) in Mplus, version 7.3. In addition, Mplus was used to conduct mediation analyses. In order to test our hypotheses we conducted an overall structural equation model including every measurement model described above. Current symptoms of MD as outcome variable was regressed on PTSD symptoms (mediator variable) and CM (independent variable) and also PTSD symptoms on CM. MD symptoms were also regressed on the proposed mediators representing emotion regulation difficulties, depressogenic attributional style and attachment, as well as on the independent variable CM. All mediators were again regressed on CM. To assess the influence of specific emotional, cognitive and interpersonal deficits on MD symptoms via PTSD symptoms, PTSD symptoms were furthermore regressed on the mediator variables. Figure 1 shows a simplified version of the computed structural equation model.

For all pathways, standardized direct, specific indirect, total indirect and total effects were estimated. The residual correlations between the postulated latent mediator variables (except from PTSD) were freely estimated. Estimation of residual correlations between PTSD symptoms and the other mediators was not necessary as we modeled direct effects of each moderator on PTSD symptoms. SEM-based models were calculated using nonlinear two parameter normal ogive item-response-theory (IRT) of probit regression for binary and ordered polytomous items. Conservatively assuming ordinality of all indicators, we used the robust weighted least squares means and variance (WLSMV) adjusted estimator (e.g. [54]), which is robust also to not normally distributed data. Only standardized values will be reported for all estimations. Missing data (only present for CTQ-Data) was estimated using full information maximum likelihood [54].

Goodness of fit was assessed with the following fit indices: Chi-square (χ^2^), comparative fit index (CFI), Tucker-Lewis index (TLI) and root mean square error of approximation (RMSEA). Thresholds were considered as follows: for CFI and TLI excellent fit >.95 and moderate fit >.90 [55]; for RMSEA excellent fit <.05 and moderate fit <.08 [56]. Due to the dependency on model complexity and sample size [57], χ^2^-values will only be reported for completeness.

## Results

### Sample Statistics

16.3% of participants met DSM-5 criteria for a current episode of major depression (categorical assessment via PHQ-9). Participants’ total scores on the PHQ-9 (dimensional assessment) indicated an even higher prevalence of depressive symptoms with 502 (48.8%) participants suffering from mild to severe depressive symptoms. Nearly half of the sample (48.7%) also reported at least one former episode of MD. The mean total score of PCL-5 was 14.22 (*SD* = 13.70) and 12.2% of participants scored above the cut-off value of 33 that is indicative of probable PTSD [52]. The mean CTQ-score was 42.82 (*SD* = 15.55). When differentiating between the postulated subtypes of childhood trauma [40], we found the following rates of severe to extreme forms of childhood trauma: 17% emotional abuse; 6.5% physical abuse; 5.9% sexual abuse; 18.4% emotional neglect and 7.1% physical neglect. More information about clinical and demographic sample characteristics is provided in Table 1.

**Table 1:** Sample Characteristics

Demographic characteristics					
Age (years): *M* (*SD*)			45.10 (11.89)		
Gender (Female): *n* (%)			705 (68.60)		
Education: *n* (%)					
- Less than high-school diploma			355 (34.60)		
- High-school diploma (or equivalent)			648 (63.10)		
- University degree			22 (2.10)		
- No degree			2 (0.20)		
Relationship Status (*n* (%))					
- In partnership			700 (68.16)		
- Divorced/Widowed			118 (11.49)		
- Not in a relationship			209 (20.35)		
Clinical Characteristics					
Current MDE according to DSM-5: *n* (%)^a^			167 (16.30)		
Severity of current depressive symptoms: *n* (%)^b^					
- none			42 (4.10)		
- minimal – mild			822 (80.00)		
- moderate – severe			163 (15.90)		
Former MDE: *n* (%)			500 (48.70)		
PCL-5 Total Score: *M* (*SD*)			14.22 (13.70)		
PCL-5 Total Score > Cut-off 33: *n*(%)^c^			125 (12.20)		
CTQ Total-Score: *M* (*SD*)			42.84 (15.55)		
CTQ-Subscales: *n* (%))	Emotional Abuse	Physical Abuse	Sexual Abuse	Emotional Neglect	Physical Neglect
None to minimal^d^	486 (47.30)	781 (76.00)	764 (74.40)	399 (38.80)	657 (64.00)
Low to moderate^d^	275 (26.80)	94 (9.20)	95 (9.30)	311 (30.30)	175 (17.00)
Moderate to severe^d^	91 (8.90)	85 (8.30)	107 (10.40)	128 (12.50)	122 (11.90)
Severe to extreme^d^	175 (17.00)	67 (6.50)	61 (5.90)	189 (18.40)	73 (7.10)

*Note. MDE* Major Depressive Episode, *PCL* PTSD Checklist for DSM-5, *CTQ* Childhood Trauma Questionnaire

^a^categorical evaluation (DSM-5 criteria) of PHQ-9

^b^dimensional evaluation (total score) of PHQ-9, classification according to Gräfe et al. [45]

^c^Cut-off score as suggested by Krüger-Gottschalk et al. [52]

^d^categories of severity as defined in the CTQ manual [63]

### Correlational Analyses

Correlations among all latent constructs are shown in Table 2. As predicted, all constructs were significantly positively associated (*p* < .001). The correlation between CM severity (independent variable) and MD symptom severity (outcome variable) was .33. The correlation coefficients between the postulated mediators and CM ranged from .13 to .47, whereby the highest correlation was found with PTSD symptoms. Associations between MD symptoms and the mediators ran between .34 and .74.

**Table 2:** WLSMV-Estimated Correlations* Between Variables of Interest (*N*=1027)

		1.	2.	3.	4.	5.	6.	7.	8.	9.	10.	11.	12.	13.
1.	CM	-	.33	.47	.27	.27	.27	.15	.24	.24	.22	.29	.33	.13
2.	MD		-	.68	.54	.65	.57	.46	.74	.63	.60	.70	.34	.44
3.	PTSD			-	.49	.53	.53	.28	.57	.46	.55	.56	.28	.39
4.	NOA				-	.60	.62	.37	.75	.58	.74	.66	.25	.45
5.	GOA					-	.74	.30	.81	.55	.62	.66	.23	.47
6.	IMP						-	.32	.76	.56	.59	.60	.20	.47
7.	AWA							-	.46	.71	.36	.44	.41	.14
8.	STR								-	.65	.75	.83	.29	.53
9.	CLA									-	.57	.60	.39	.35
10.	RUM										-	.76	.24	.52
11.	DA											-	.35	.53
12.	AVO												-	.12
13.	ANX													-

*Note.* *standardized correlation coefficients; all correlations were found to be significant at the *p*<.001 level. *CM* Childhood Maltreatment, *MD* Symptoms of Major Depression, *PTSD* Posttraumatic Stress Disorder symptoms, *NOA* Nonacceptance of emotional responses, *GOA* Difficulties engaging in goal-directed behavior, *IMP* Impulse control difficulties, *AWA* Lack of emotional awareness, *STR* Limited access to emotion regulation strategies, *CLA* Lack of emotional clarity, *RUM* Rumination, *DA* Depressogenic Attributions, *AVO* Avoidance, *ANX* Anxiety

### Mediational Analysis

Our first hypothesis stated that the calculated sequential mediation model (see Fig. 1) would show a good model fit. This was confirmed by the fit-indices for this model, χ^2^(8958) = 19458.37, *p* < .001, RMSEA = .03, CFI = .92, TLI = .92. More specifically, the RMSEA was excellent, and the CFI und TLI showed a moderate fit. In addition, the model explained a high proportion of variance in MD symptomatology (*R*^*2*^ = .69, *p* < .001).

In line with the second hypothesis, a mediation of the association between CM and MD symptoms through the postulated mediators was found. The total indirect effect was highly significant (β = .32; *p* < .001), while there was no significant direct effect from CM to MD symptoms anymore in the total model (β = .002, *p* = .957).

In order to test Hypothesis 3, the specific indirect effects were inspected. As predicted, emotion regulation deficits, depressogenic attributional style and PTSD symptom severity were all found to be significant mediators (Hypotheses 3a and 3b). We also found a significant indirect effect for the mediator *rumination*, as a facet of emotion dysregulation, to MD symptoms via PTSD symptoms (Hypothesis 3c). Among the different indices of emotion regulation difficulties, the facets *limited access to emotion regulation strategies* (β = .09, *p* < .001) and *lack of emotional clarity* (β = .04, *p* < .01) showed the largest effects. The indirect effect of depressogenic attributional style was β = .04 (*p* < .01), and PTSD symptoms was the strongest mediator with an indirect effect of β = .11 (*p* < .001). The indirect effect of rumination on the association of CM and MD symptoms via PTSD symptoms was β = .01 (*p* < .05). All total, direct, total indirect and specific indirect effects are displayed in Table 3.

**Table 3:** Total, Direct, Total Indirect and Specific Indirect Effects

Effects from	Total (95% CI)	Direct (95% CI)	Total indirect (95% CI)
CM to MD	.33*** (.255, .395)	.00 (-.060, .064)	.32*** (.264, .383)
Mediating variable (M)	Effect of CM on M (95% CI)	Effect of M on MD (95% CI)	Specific Indirect Effect (95% CI)
PTSD	.31*** (.238; .372)	.34*** (.268; .419)	.11*** (.072, .137)
NOA	.21*** (.138; .274)	-.11* (-.198; -.015)	-.02* (-.042, -.002)
GOA	.27*** (.204; .332)	.11* (.012; .209)	.03* (.002, .057)
IMP	.27*** (.199; .339)	-.10* (-.196; -.009)	-.03* (-.054, -.001)
AWA	.15*** (.082; .220)	.04 (-.041; .115)	.01 (-.006, .018)
STR	.24*** (.174; .307)	.36*** (.202; .519)	.09*** (.042, .132)
CLA	.24*** (.171; .302)	.18*** (.080; .274)	.04** (.017, .067)
RUM	.22*** (.146; .291)	-.03 (-.136; .076)	-.01 (-.030, .017)
DA	.29*** (226; .355)	.14** (.041; .245)	.04** (.011, .072)
AVO	.33*** (.270; .390)	.03 (-.018; .085)	.01 (-.006, .028)
ANX	.13*** (.066; .200)	.03 (-.035; .086)	.00 (-.005, .012)
	Effect of M on PTSD (95% CI)	Specific Indirect Effect (95% CI)	
NOA (via PTSD)	.00 (-.113;.115)	.00 (-.008, .008)	
GOA (via PTSD)	.07 (-.052; .194)	.01 (-.005, .018)	
IMP via PTSD	.11 (-.010; .219)	.01 (-.002, .021)	
AWA via PTSD	-.05 (-.140; .049)	.00 (-.007, .003)	
STR via PTSD	.08 (-.106; .271)	.01 (-.009, .022)	
CLA via PTSD	.09 (-.021; .209)	.01 (-.002, .017)	
RUM via PTSD	.18** (.045; .323)	.01* (.002, .025)	
DA via PTSD	.08 (-.059; .210)	.01 (-.006, .021)	
AVO via PTSD	.02 (-.041; .084)	.00 (-.005, .010)	
ANX via PTSD	.06 (-.015; .126)	.00 (-.001, .006)	

*Note.* As many lower bounds of reported confidence intervals are close to zero, three digits after the decimal point are reported to avoid confusion.

*CI* confidence interval, *CM* Childhood Maltreatment, *MD* Symptoms of Major Depression, *PTSD* Posttraumatic Stress Disorder symptoms, *NOA* Nonacceptance of emotional responses, *GOA* Difficulties engaging in goal-directed behavior, *IMP* Impulse control difficulties, *AWA* Lack of emotional awareness, *STR* Limited access to emotion regulation strategies, *CLA* Lack of emotional clarity, *RUM* Rumination, *DA* Depressogenic Attributions, *AVO* Avoidance, *ANX*

*** *p*<.001; ** *p*<.01; * *p*<.05

## Discussion

Understanding psychological mediators of the association between CM and symptoms of MD appears essential to identify appropriate targets for novel treatment approaches that can be offered to CM survivors with MD symptoms. Prior research has found emotional, cognitive and attachment deficits as well as PTSD symptoms to mediate between CM and MD symptoms (e.g. [20–22, 26]). Our study aimed to replicate and extend previous findings on direct and indirect effects of childhood CM on later symptoms of MD, conducting a SEM-based sequential mediation model within a large community sample.

As predicted, CM and MD symptoms were substantially positively associated, in that higher levels of CM corresponded with more severe depressive symptoms. This is in line with recent meta-analytic results [4, 5].

The sequential mediation model proposed a priori (see Fig. 1) showed a good model fit, supporting our first hypothesis. In contrast to prior studies (e.g. [20–22]) but in line with our second hypothesis, we found a mediation of the association of CM and MD symptoms with a substantial and significant indirect, but no remaining direct effect of CM on MD symptoms. Importantly, the total indirect effect had two main sources: PTSD symptom severity as a strong mediator between CM and MD symptoms on the one hand, and dysfunctional cognitive and emotional processes (i.e., ER difficulties, depressogenic appraisals) on the other hand. Only one variable was found to sequentially mediate the association between CM and MD symptoms via PTSD symptom severity, namely rumination. No indirect effect was found for attachment-related anxiety or avoidance.

It should be noted that the specific indirect effects were mostly in the small range and that all mediators were highly correlated. Therefore, no strong conclusions about the relative importance of one of these mediators in comparison to another can be drawn. The interpretation of findings should mainly focus on the overall pattern of mediation, not single pathways. In our view, the good fit of the overall model, the evidence for the mediational effects on the association between CM and MD symptoms by the psychological variables specified in the model, and the high amount of variance of MD symptom severity explained by the model (69%) generally supports the validity of the theoretical assumptions underlying the current study. Our study supports the general relevance of emotional and cognitive processes as well as posttraumatic stress symptoms when investigating the association of CM and symptoms of MD, but cannot lead to final conclusions about mediation processes. Specifically, results are firstly in line with the idea that CM impairs the development of self-regulation on an emotional, cognitive and possibly also interpersonal level, resulting e.g. in poor ER, and a depressogenic attributional style. Importantly, these cognitive and emotional sequelae of CM then increase the risk for a later development of MD symptoms (see e.g. [21, 22] for similar findings). However, our results secondly show that PTSD symptoms are an important additional mediator between CM and MD symptoms that has only rarely been included in earlier research (for an exception, see [22]). The strong mediational role of PTSD symptoms may suggest that the trauma of CM itself has not been processed in an adaptive way, leading to intrusive re-experiencing, hyperarousal and/or high emotional and physiological responding to trauma reminders, which in turn leads to symptoms of depression.

In summary, our results reveal the potential influence of our postulated mediators and their relevance for future research. Yet, to draw specific conclusions further investigations are strongly required. Only if replicated in future studies, the findings may have important clinical implications. Importantly, they suggest promising targets for novel treatments that could be offered to CM survivors suffering from MD symptoms. First, treatments for MD following CM may need to include strategies aimed at improving cognitive and emotional self-regulation. However, more research is needed to identify the specific processes that should be targeted. Second, for CM survivors suffering from depression who additionally show high levels of CM-related memories, thoughts, feelings, or behaviors, trauma-focused treatments aimed at processing the traumatic experience may be promising. Whereas trauma-focused treatment can be regarded as state-of-the art for CM survivors suffering from PTSD (e.g. [58]), a key question for future research will be to test whether trauma-focused treatment is also indicated for CM-related MD without full-blown PTSD but with subclinical signs of CM-related memories, thoughts or feelings.

## Strengths and Limitations

Our study shows a number of strengths, including the large sample size, the simultaneous testing of several mediators within one sequential mediation model, and the use of SEM. On the other hand, limitations need to be taken into account when interpreting our findings. First, the current study used a cross-sectional design, which clearly does not allow the establishment of temporal precedence of the mediators. We think that a cross-sectional design is defendable as a first step; however, future research using longitudinal designs are clearly warranted to draw causal conclusions. Moreover, our mediational model was derived a priori from previous research. However, it is of course only one of several possible and reasonable models on how the different variables of interest may be related. Further validation of our findings would require the comparison of our model against others which for example include MD as mediator and PTSD as dependent variable. Second, CM was assessed retrospectively, which can be biased by current states, including current symptoms of depression [59, 60]. Reassuringly, however, Scott et al. [59] could not find significant differences between retrospective and prospective ascertainment of CM. Also, we cannot exclude that a positive response bias might have influenced our results such as the high correlations of interesting variables. Although we aimed to test a community sample, descriptive analyses revealed higher rates of psychopathology and trauma exposure in our sample than in the normal German population (e.g., point-prevalence of 16.3% for current MDE; CTQ mean score: 42.82; cf. [42, 61, 62]). Besides, our sample consisted of registered users of an online panel. Users registering for the panel declare that they are interested in supporting psychological research. This preselection is a possible source for bias. Moreover, almost a third of our participants discontinued the survey. By definition, these participants did not provide final consent for data analysis at the end of the survey. However, as this is a requirement for analyzing data from online studies set by our local ethics committee, we were unable to test whether participants in our final sample differed from participants who discontinued the survey. Due to possible selection bias both during recruitment and (dis-) continuation of the study, our sample cannot be regarded as representative for the general population. Therefore, the generalizability of our results to other samples needs to be tested in future studies. Our sample also showed higher levels of both childhood maltreatment and depressive symptoms than to be expected in a non-clinical sample. Although this is another aspect, in which our sample differs from a general population samples, it can be argued that oversampling of individuals with high levels of depression and/or high levels of CM increases the variance of interesting constructs and can consequently be seen as favorable to test our mediation model. Finally, it has to be mentioned that our model does not control for the influence of possible confounding variables like age, gender, socio-economic status. Although our results support the general relevance of postulated mediators, the validity in more specified samples has to be investigated in future studies.

## Conclusions

Our study replicates and extends previous results, showing that the association of CM and MD can be explained when incorporating ER, depressogenic attributions, attachment styles and symptoms of PTSD as mediators. If replicated in future research, clinical implications may include both the development of trauma-focused interventions aiming at processing traumatic memories for CM survivors suffering from chronic depression, as well as strategies to improve cognitive and emotional self-regulation in MD patients who have been affected by CM.

## Abbreviations

CFA: Confirmatory factor analyses
CFI: Comparative fit index
CI: Confidence interval
CM: Childhood Maltreatment
CTQ: Childhood Trauma Questionnaire
DAQ: Depressive Attributions Questionnaire
DERS: Difficulties in Emotion Regulation Scale
DSM: Diagnostic and Statistical Manual for Mental Disorders
ECR: Experiences in Close Relationship Scale
ER: Emotion Regulation
IRT: Item-response-theory
M: Mean
MD: Major depressive disorder
PCL: PTSD-Checklist
PHQ: Patient Health Questionnaire
PTSD: Posttraumatic stress disorder
RMSEA: Root mean square error of approximation
RSQ: Response Styles Questionnaire
SCID: Structured Clinical Interview
SD: Standard deviation
SEM: Structural equation modelling
TLI: Tucker Lewis index
WLSMV: Weighted least square means and variance

## Footnotes

1 Of note, informed consent was asked both at the beginning and at the end of the survey. Consequently, dropouts due to discontinuance cannot be analyzed.

2 Within the remaining cases, the percentage of missing items indicative for the CTQ was very low (0.48%). The highest percentage of missings (0.65%) was found among the indicator items of the subscale *emotional neglect.*
